# Supplementary material for: Serum proteomics reveals disorder of lipoprotein metabolism in sepsis
Source: Life Sci Alliance. 2021 Aug 24;4(10):e202101091. doi: 10.26508/lsa.202101091 (PMC8385306; doi:10.26508/lsa.202101091)
Supplement: Supplementary file 1 [file LSA-2021-01091_TableS1.docx]

**Table S1** Characteristics of enrolled patients and NC subjects included in the derivation group.

|  | Sepsis | NC | p |
| --- | --- | --- | --- |
| n | 59 | 31 |  |
| Male (%) | 33 (55.9%) | 19 (61.3%) | 0.707 |
| Age (years) | 70.50 [61.0, 78.0] | 68.0 [65.0, 70.50] | 0.870 |
| Laboratory data |  |  |  |
| Mean arterial pressure (mm Hg) | 79.3 [71.2, 89.8] | NA | NA |
| White blood cell count (10^9^/L) | 12.8 [7.6, 18.4] | 5.4 [4.5, 6.5] | <0.01 |
| Haemoglobin (g/L) | 114.5 [92.7, 127.] | 149.0 [139.5, 160.0] | <0.01 |
| Haematocrit (%) | 33.9 [28.2, 38.2] | 44.5 [40.3, 47.6] | <0.01 |
| Platelet count (10^9^/L) | 136.0 [68.2, 198.7] | 226.0 [190.5, 236.5] | <0.01 |
| Albumin (g/dL) | 27.1 [24.9, 30.1] | 45.50 [43.70, 46.0] | <0.01 |
| Aspartate aminotransferase (U/L) | 45.5 [26.2, 122.2] | 21.0 [18.5, 25.5] | <0.01 |
| Alanine aminotransferase (U/L) | 28.5 [17.5, 66.8] | 20.0 [14.5, 24.5] | <0.01 |
| Total bilirubin (μmol/L) | 15.6 [7.8, 26.2] | 16.5 [12.2, 19.0] | 0.688 |
| Creatinine (μmol/L) | 135.0 [95.0, 218.0] | 60.0 [52.5, 76.0] | <0.01 |
| INR | 1.2 [1.1, 1.3] | NA | NA |
| Infection |  |  |  |
| Gram-positive bacteria (%) | 1 (4.2%) | NA | NA |
| Gram-negative bacteria (%) | 9 (37.5%) | NA | NA |
| Viral (%) | 1 (4.2%) | NA | NA |
| Other (%) | 13 (54.1%) | NA | NA |
| CRRT | 8 (13.6%) | NA | NA |
| Vasopressors |  | NA | NA |
| No (%) | 30 (50.8%) |  |  |
| Yes (%) | 25 (42.4%) |  |  |
| Unknown (%) | 4 (6.8%) |  |  |
| Mechanical ventilation |  | NA | NA |
| No (%) | 30 (50.8%) |  |  |
| Yes (%) | 22 (37.3%) |  |  |
| Unknown (%) | 7 (11.9%) |  |  |
| Severity at time of admission to ICU |  |  |  |
| SOFA | 6.0 [4.0, 9.0] | NA | NA |
| APACHE II | 18.5 [11.0, 22.0] | NA | NA |
| Mortality |  |  |  |
| 28-day | 7 (11.9%) | NA | NA |
| 90-day | 7 (11.9%) | NA | NA |

Data are expressed as the mean ± SD, median (IQR) or number of patients (percentages). Continuous variables were compared by using Student’s t-test and the Mann-Whitney U test, and categorical variables were compared by using the χ2 or Fisher’s exact test between the derivation and validation groups.

APACHE II=Acute Physiology and Chronic Health Evaluation II. SOFA=Sequential Organ Failure Assessment on day of sampling.
