# Supplementary material for: Serum proteomics reveals disorder of lipoprotein metabolism in sepsis
Source: Life Sci Alliance. 2021 Aug 24;4(10):e202101091. doi: 10.26508/lsa.202101091 (PMC8385306; doi:10.26508/lsa.202101091)
Supplement: Supplementary file 4 [file LSA-2021-01091_TableS4.docx]

**Table S4** The information of ELISA kits used in this study.

| Kits | Source | Cat. No. |
| --- | --- | --- |
| RayBio^®^ Human CRP (C-Reactive Protein) ELISA Kit | RayBiotech, Norcross, GA | ELH-CRP-1 |
| RayBio^®^ Human LBP (Lipopolysaccharide-Binding Protein) ELISA Kit | RayBiotech, Norcross, GA | ELH-LBP-1 |
| RayBio^®^ Human LRG1 (Leucine-rich alpha-2-glycoprotein) ELISA Kit | RayBiotech, Norcross, GA | ELH-LRG1-1 |
| RayBio^®^ Human SAA (Serum Amyloid A-1 protein) ELISA Kit | RayBiotech, Norcross, GA | ELH-SAA-1 |
